# Supplementary material for: Extraction of coronary thrombus-derived exosomes from patients with acute myocardial infarction and its effect on the function of adventitial cells
Source: PLoS One. 2025 Jan 16;20(1):e0313582. doi: 10.1371/journal.pone.0313582 (PMC11737788; doi:10.1371/journal.pone.0313582)

Original Image for Figure2 C

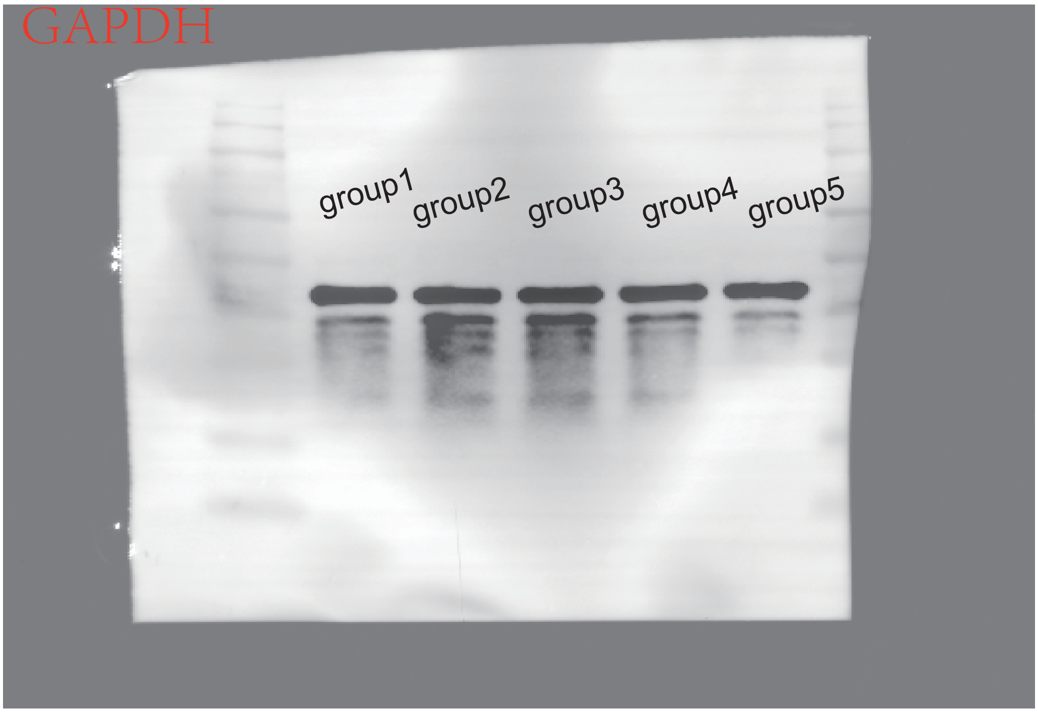

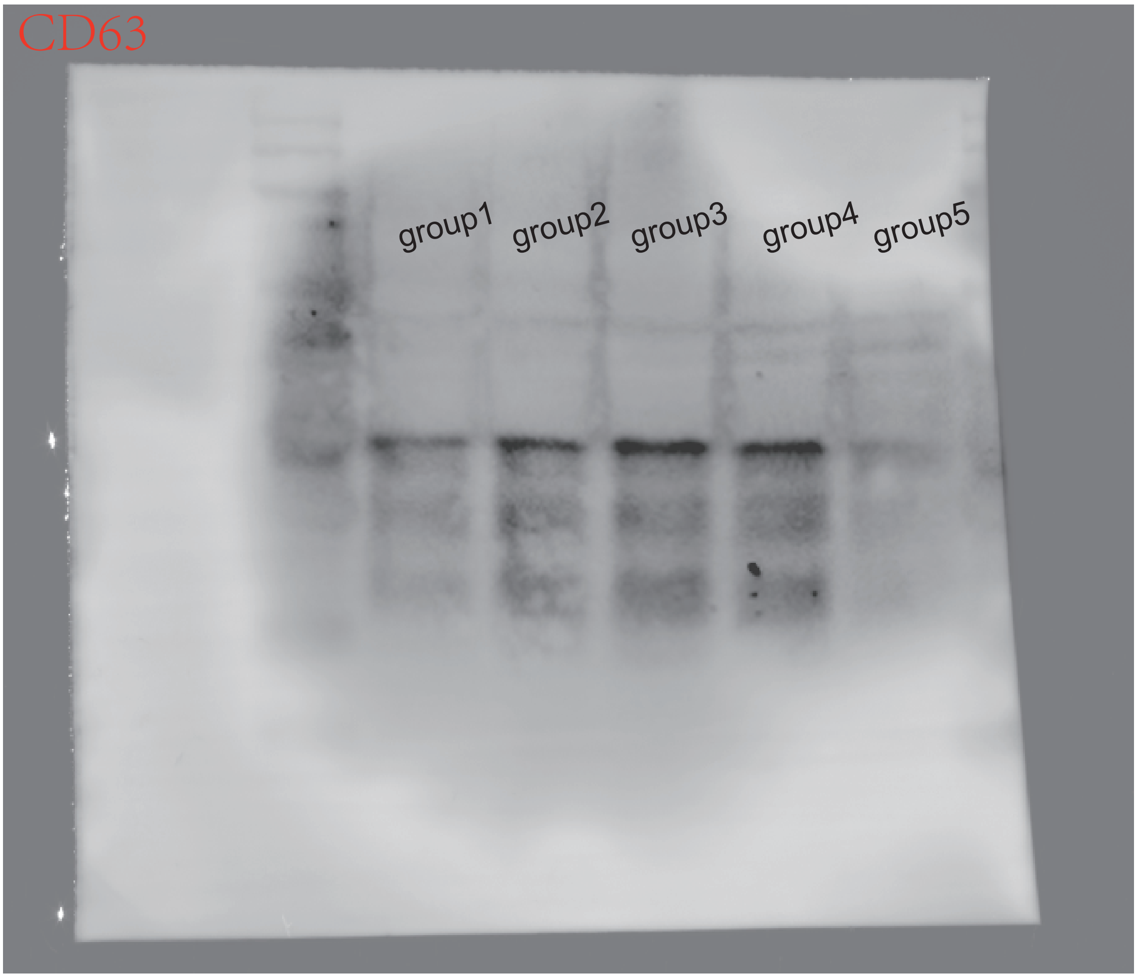

Original Image for Figure3 C

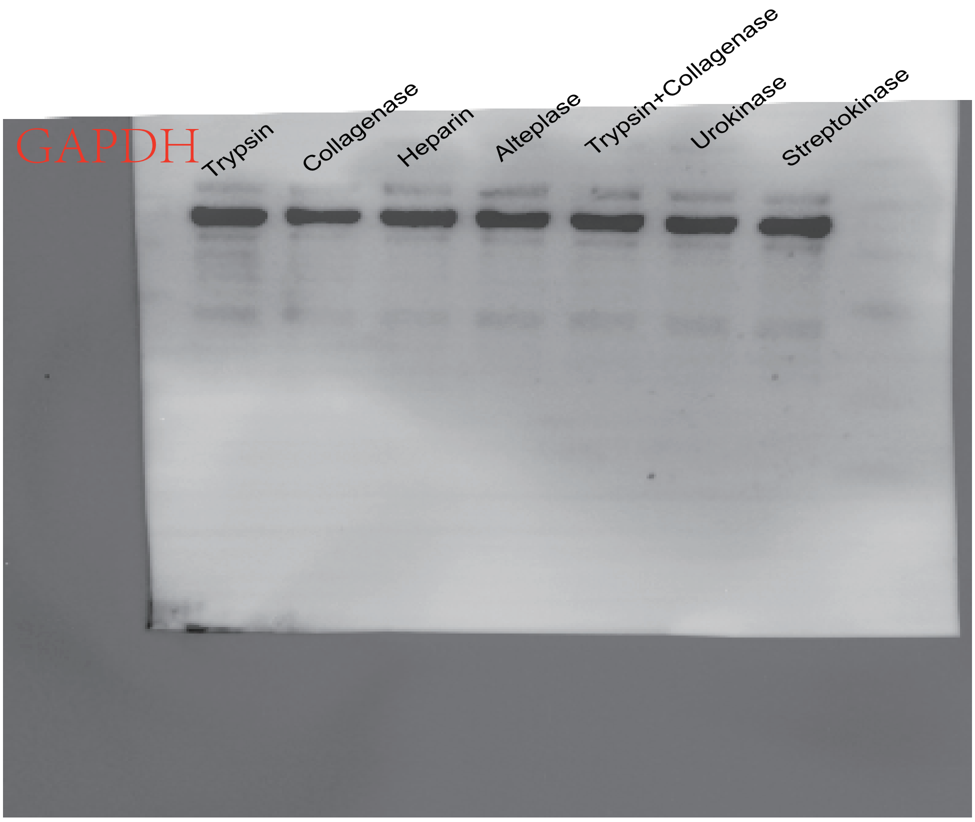

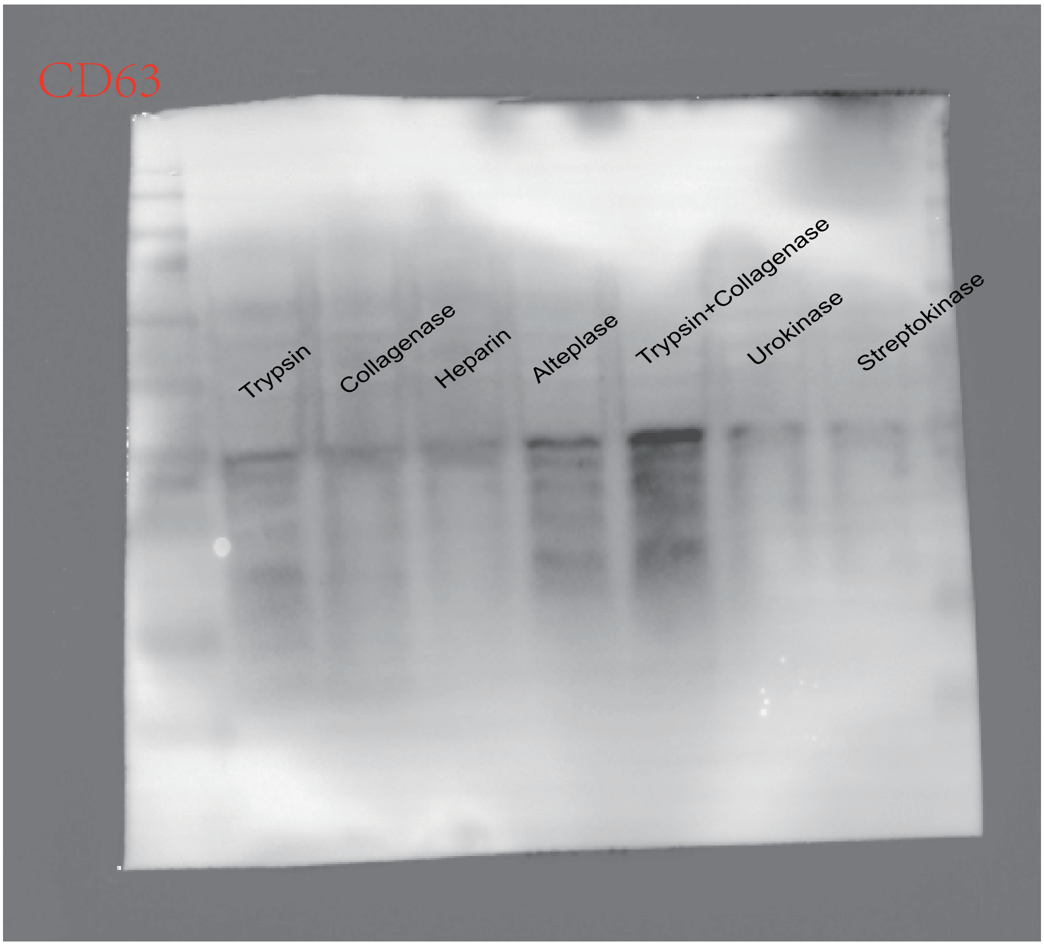

Control HSE ASE TE

Original Image for Figure5 E

GPX4

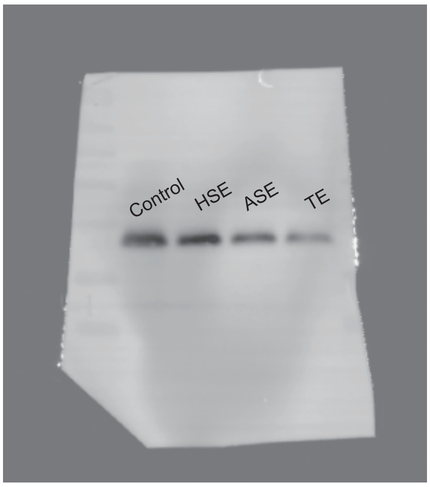

caspase8

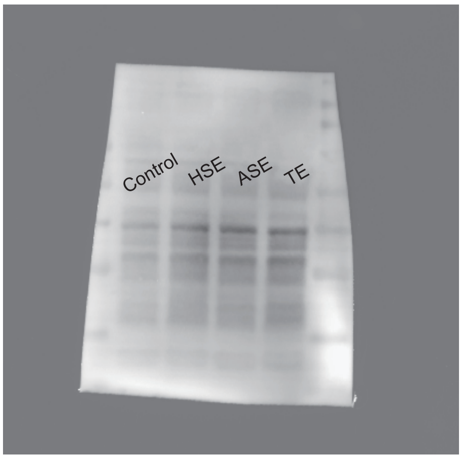

caspase9

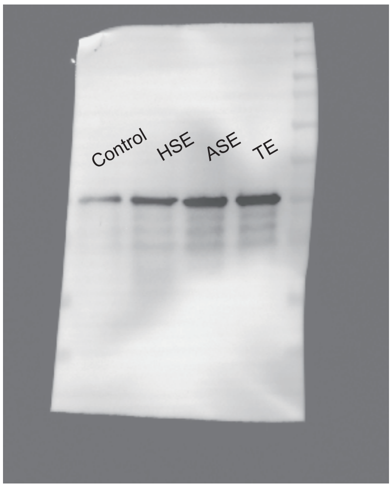

cleaved-caspase3

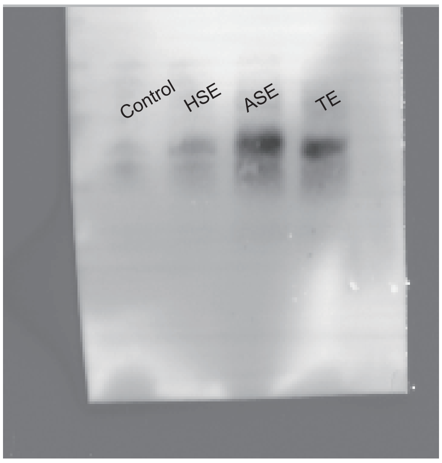

P62

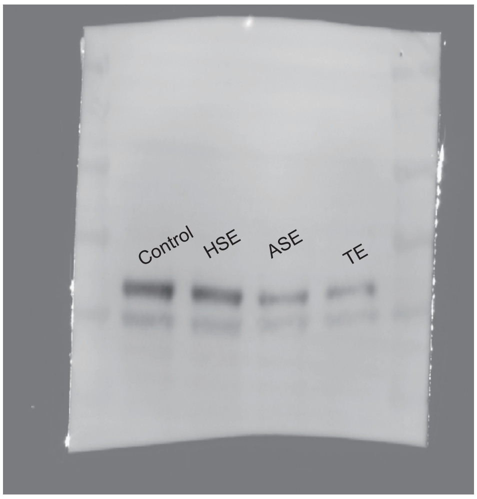

LC3

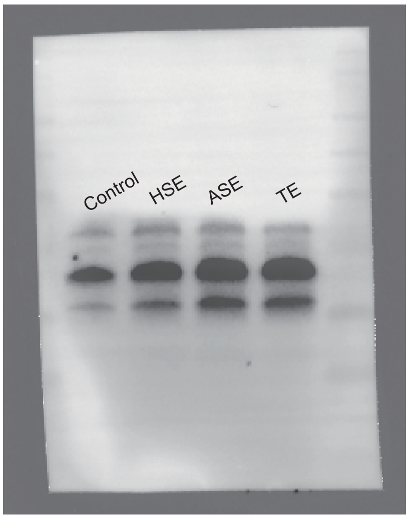

GAPDH

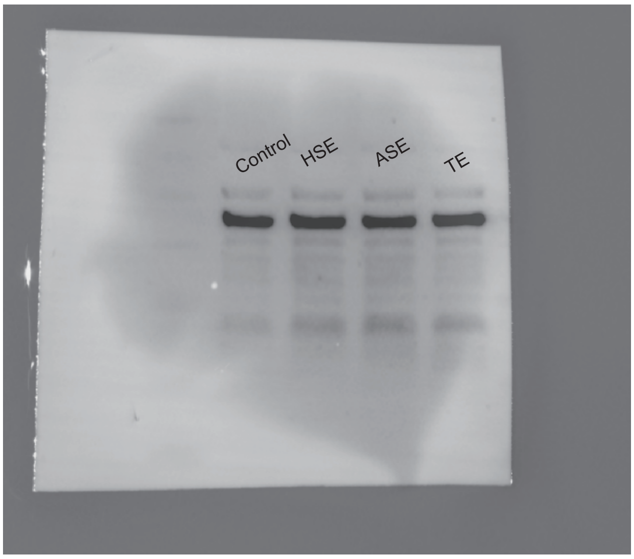

Original Image for Figure6 B

GAPDH

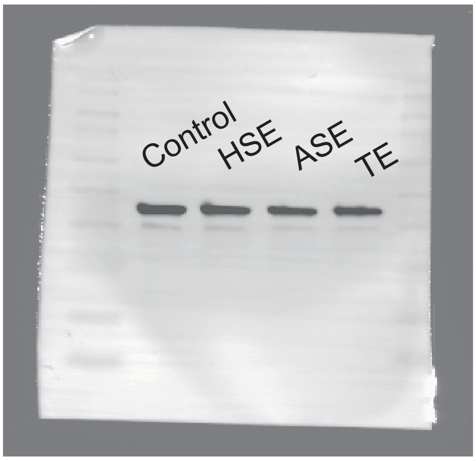

CyclinD1

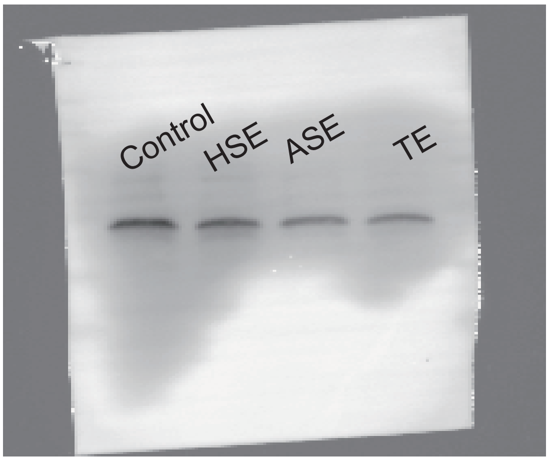

PCNA

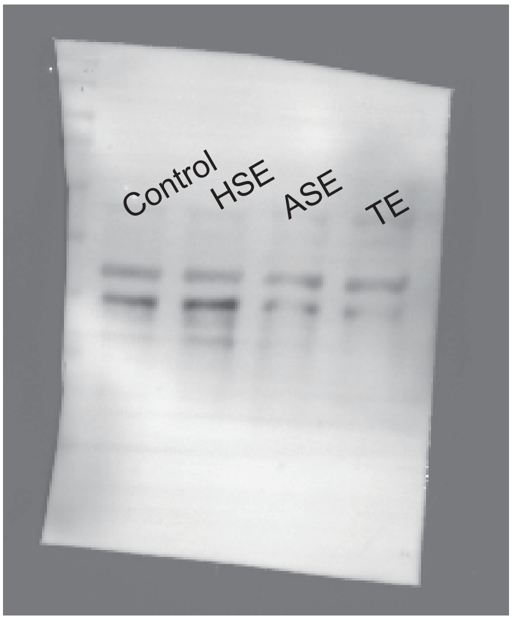

cleaved-caspase3

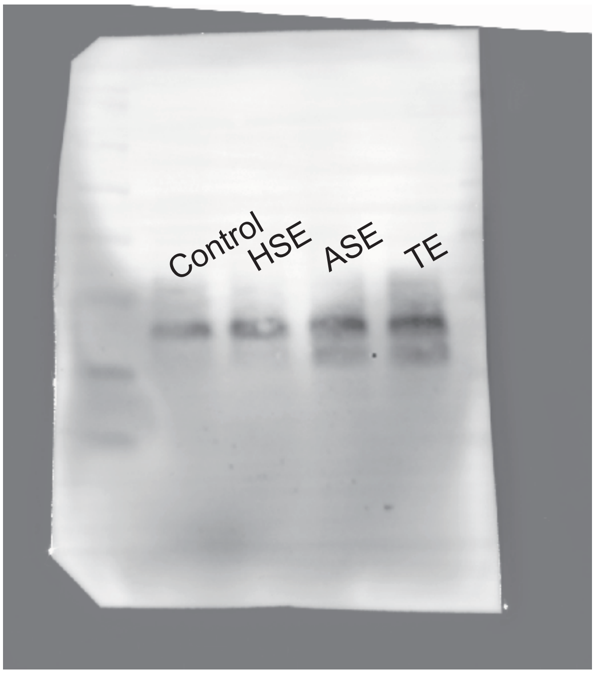

Original Image for Figure7 B

GAPDH

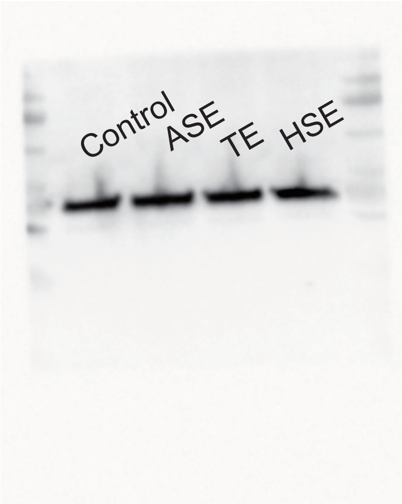

CyclinD1

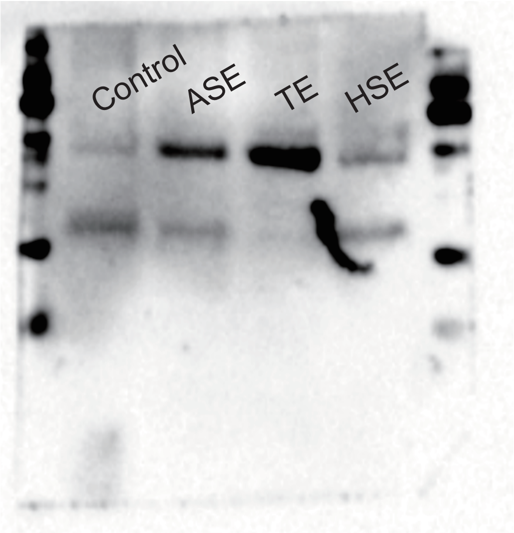

PCNA

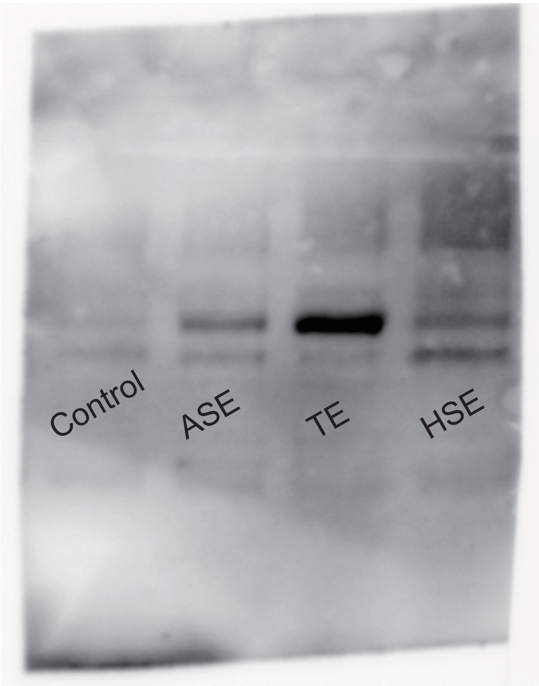

OPN

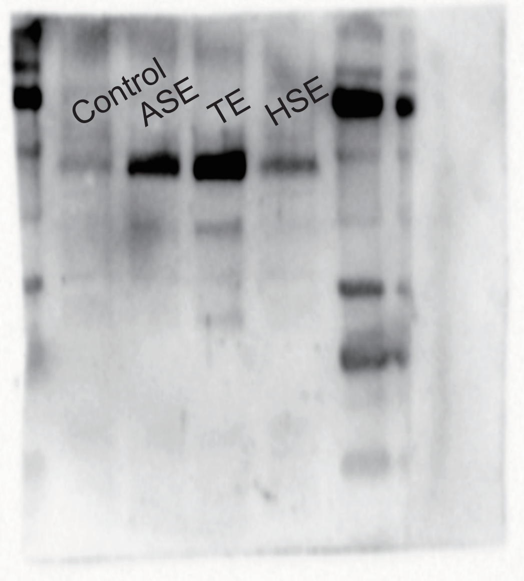

α -SMA

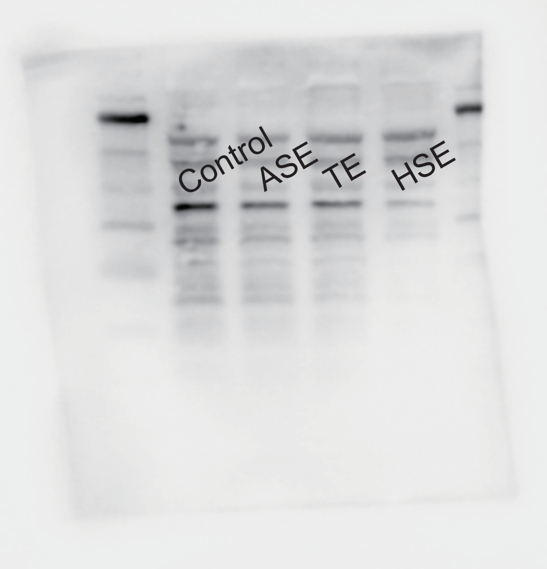

Supplement: S1 Raw images — (PDF) [file pone.0313582.s002.pdf]
